# Genome-Scale Identification, Classification, and Expression Profiling of MYB Transcription Factor Genes in *Cinnamomum camphora*

**DOI:** 10.3390/ijms232214279

**Published:** 2022-11-18

**Authors:** Xiaoyue Luan, Wenlin Xu, Jiaqi Zhang, Tengfei Shen, Caihui Chen, Mengli Xi, Yongda Zhong, Meng Xu

**Affiliations:** 1 Co-Innovation Center for Sustainable Forestry in Southern China, Key Laboratory of Forest Genetics and Biotechnology of Ministry of Education, Nanjing Forestry University, Nanjing 210037, China; 2 Key Laboratory of Horticultural Plant Genetics and Improvement of Jiangxi Province, Institute of Biological Resources, Jiangxi Academy of Sciences, Nanchang 330096, China

**Keywords:** *Cinnamomum camphora*, *CcMYB*, MYB transcription factors, camphor genome, gene expression

## Abstract

The camphor tree (*Cinnamomum camphora* (L.) Presl.) is the representative species of subtropical evergreen broadleaved forests in eastern Asia and an important raw material for essential oil production worldwide. Although MYBs have been comprehensively characterized and their functions have been partially resolved in many plants, it has not been explored in *C. camphora*. In this study, 121 *CcMYBs* were identified on 12 chromosomes in the whole genome of *C. camphora* and found that *CcMYBs* were mainly expanded by segmental duplication. They were divided into 28 subgroups based on phylogenetic analysis and gene structural characteristics. In the promoter regions, numerous *cis*-acting elements were related to biological processes. Analysis of RNA sequencing data from seven tissues showed that *CcMYBs* exhibited different expression profiles, suggesting that they have various roles in camphor tree development. In addition, combined with the correlation analysis of structural genes in the flavonoid synthesis pathway, we identified *CcMYBs* from three subgroups that might be related to the flavonoid biosynthesis pathway. This study systematically analyzed *CcMYBs* in *C. camphora*, which will set the stage for subsequent research on the functions of *CcMYBs* during their lifetime and provide valuable insights for the genetic improvement of camphor trees.

## 1. Introduction

The V-Myb avian myeloblastosis viral oncogene family, known as MYB transcription factors (TFs), comprises a large superfamily in plants and is characterized by highly conserved DNA-binding domain repeats [1]. MYB proteins can be divided into four subfamilies according to their number of repeats. Those containing only one or part of the repeat, called 1R-MYB or MYB-related proteins [2], mediate the oscillation of target genes involved in secondary metabolism, cell and organ morphogenesis, antioxidant defense systems, and regulation of various signaling pathways [3,4,5,6]. The R2R3-MYB subfamily, which contains two repeats, has the largest number of MYB members. R2R3-MYB proteins may have evolved through the R1 deletion of 3R-MYB [2] and are related to plant secondary metabolism, growth and development, and various abiotic stresses [7,8]. The 3R-MYB subfamily is composed of three adjacent repeats that are highly homologous to the 3R-MYB protein in animals and fungi. It participates in the control of the cell cycle and regulates cell differentiation by recognizing M-specific activator (MSA) elements and regulating G2/M phase transcription. The smallest subfamily is 4R-MYB, consisting of four R1/R2-like repeats, which have been found in *Homo sapiens*, *Drosophila melanogaster*, and some plants [9,10,11,12]. Some studies have found that 4R-MYBs are essential for gametophyte and zygote development [10]. Previous research has shown that the size of *MYBs* in plants is mainly due to the rapid expansion of the *R2R3-MYB* subfamily [1,13].

MYB proteins are widely involved in various biological processes, including cell differentiation, cell cycle regulation, responses to abiotic stresses, and secondary metabolite synthesis [1,5,14]. Abiotic stress can adversely affect plant growth. MYB TFs have been reported to participate in the response of *Arabidopsis thaliana* (L.) Heynh to biotic and abiotic stresses. AtMYB30 can be targeted by the *Xanthomonas* type III effector XopD, which suppresses AtMYB30-mediated plant defense, showing the critical role of AtMYB30 in the regulation of plant disease resistance [15]. The overexpression of *AtMYB60*, *AtMYB96,* and *AtMYB44* proved that they enhanced the plants sensitivity to drought stress [16,17,18]. In apple plants (*Malus sieversii* f. *niedzwetzkyana*), MdMYB94 can improve tolerance to water-deficit stress and promote waxy biosynthesis in the leaf cuticle [19]. Additionally, MYB TFs can participate in regulating secondary metabolites, which can assist plants in adapting to changing environments. Flavonoids (bioflavonoids) are polyphenolic secondary metabolites found in the vacuoles of plant cells. They are the most important plant pigments for flower coloration and play an important role in signaling and light protection [20]. MYB TFs can regulate the expression of structural genes that affect flavonoid accumulation. GMYB10 specifically promotes the enrichment of cyanidin in callus and vegetative tissues by activating DFR and increasing pelargonidin content in the stamens of *Gerbera hybrida* [21,22]. GtMYB3 facilitates the accumulation of delphinidin in petals by inducing the expression of the *F3’5’H* gene in *Gentiana trifloral* [23]. *Oncidium gower* MYB TFs are widely involved in regulating anthocyanin synthesis by activating CHI and DFR, which significantly stimulates the accumulation of various pigments, such as cyanidin, delphinidin, malvidin, and pelargonidin, in petals [24]. Some MYB TFs have been shown to regulate plant organogenesis. For example, *AtMYB103*, WER, *AtMYBGL1*, and *AtMYB23* play vital roles in the development of the tapetum, trichomes, and root hairs in *Arabidopsis* [25,26].

Currently, the functions of MYB TFs have been identified in *Arabidopsis*, apples, grapevine (*Vitis vinifera ‘Shiraz’*), and other tree species [27,28,29,30,31,32], but there are few reports on camphor trees. *C. camphora* is a representative species of the genus *Cinnamomum* in the Lauraceae family and is native to eastern Asia. It is an important native and landscaping tree species in China, with a long history of cultivation. *C. camphora* plays an important role in the chemical industry because it is rich in secondary metabolites. It is an economically important tree species of the Lauraceae family and is widely distributed in southern China. In 2021, a genome sequencing of *C. camphora* was completed [33]. As an economically important tree species, *C. camphora* is rich in secondary metabolites and is commonly used as a raw material in the chemical and medicinal industries [34,35,36]. With the completion of the whole genome sequencing of *C. camphora* and the progress of omics technology [33], the synthetic pathway of essential oils and related synthases in *C. camphora*, such as the terpene synthase (*TPS*) family and the trans-isopentenyl diphosphate synthase (*TIDS*) gene family, have been analyzed [37,38,39,40]. However, a study of the camphor MYB TFs (*CcMYB*) family has not been reported.

In this study, 121 *CcMYBs* were identified based on the high-quality chromosome level of the *C. camphora* genome sequenced previously [33], and then phylogenetic analysis was performed on them and the motif composition was analyzed. We also analyzed the exon–intron distributions and duplication events of the *CcMYBs*. Using RNA-seq data, we analyzed and visualized the expression profiles of *CcMYBs* in roots, fruits, leaves, phloem, flowers, xylem, and stems of *C. camphora*. In addition, combined with correlation analysis of structural genes in the flavonoid synthesis pathway, we identified some *CcMYBs* that may have important roles in *C. camphora* growth and flavonoid synthesis. The results are not only conducive to the analysis of the functions of *CcMYB* TFs at the genome-wide level in *C. camphora* but also set the stage for subsequent research on the functions of *CcMYBs* during the lifetime of the camphor tree.

## 2. Results

### 2.1. Identification and Classification of MYBs in *C. camphora*

Based on the number of *CcMYB* domains, twenty-one 1R-MYBs (MYB-related), ninety-six R2R3-MYBs, and four 3R-MYBs were identified, but no 4R-MYBs were found (Table 1). In all, 121 *CcMYB* genes were named *CcMYB1* to *CcMYB121* according to their location on the chromosome. The *CcMYB* genes encoded proteins with amino acid residues, ranging in length from 121 aa (*CcMYB45*) to 1121 aa (*CcMYB3*), with an average length of 339. The molecular weights (MWs) ranged from 12.83 kDa (*CcMYB3*) to 113.49 kDa (*CcMYB60*), with an average of 37.14 kDa. The average theoretical isoelectric point was 6.92 and ranged from 4.93 (*CcMYB6*) to 11.16 (*CcMYB45*). The instability coefficients ranged from 29.64 for *CcMYB52* to 71.79 for *CcMYB121*. Only four proteins had instability coefficient values under 40, suggesting that most MYBs might be unstable. Grand average of hydropathicity (GRAVY) had an average value of −0.68, ranging from −0.967 (*CcMYB14*) to −0.334 (*CcMYB82*) (Table S1).

### 2.2. Conserved Domain and Phylogeny

**Figure 1:**
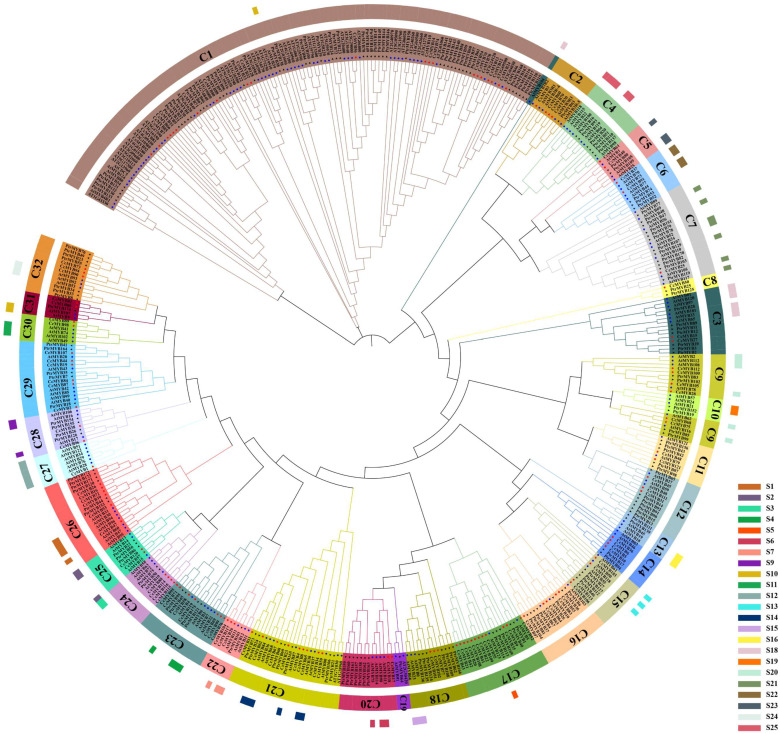
Phylogenetic analysis of MYB proteins. Phylogenetic trees of MYB proteins were constructed using MEGA X with default parameters. The 32 subgroups are indicated in different colors. Red stars represent *C. camphora* MYB proteins, blue circles represent *Arabidopsis* MYB proteins, and black triangles represent *P. trichocarpa* MYB proteins. The inner circle indicated the 32 groups, called C1–C32, of the three species, and the outer colored bars indicated the groups S1–S25 of *Arabidopsis*.

In this study, the phylogenetics of *CcMYBs* in camphor tree was analyzed with reference to 179 *A. thaliana* MYBs and 175 *Populus trichocarpa* MYBs (Table S2). The phylogenetic tree revealed that the *CcMYBs* could be divided into 28 subgroups (C4, C10, C13, and C19 subgroups did not contain *CcMYBs*), and each subgroup contained different proportions of members (Figure 1). The C1 subgroup had the most members, with 21 *CcMYBs* (17.36%), followed by the subgroup C17, which had 12 *CcMYBs* (9.92%), but C7, C8, and C27 each contained only 1 *CcMYB* (0.83%). The R2R3-MYB family in *Arabidopsis* is divided into 23 subgroups (S) based on conserved domains and accessory motifs [41]. Through the phylogenetic relationship analysis, we found that S7, S5, and S6 subgroups were clustered with the C22, C17, and C20 subgroups of camphor trees, respectively, as shown in Figure 1.

**Figure 2:**
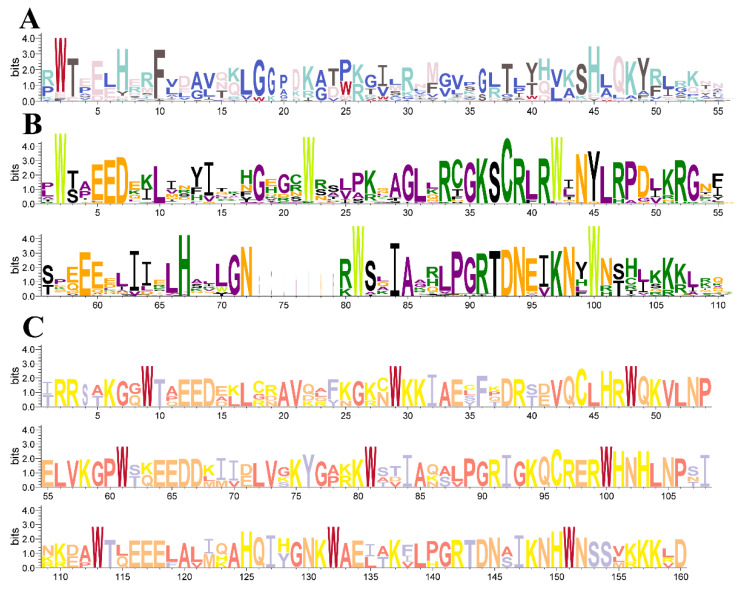
DNA-binding domains in *CcMYBs*. (**A**). The sequence logos of the 1R-MYB in *C. camphora*; (**B**). The sequence logos of the R2R3-MYB in *C. camphora*, R2 repeat in the first row, R3 repeat in the second row; (**C**). The sequence logos of the 3R-MYB in *C. camphora*, R1 repeat in the first row, R2 repeat in the second row, and R3 repeat in the third row. The bit score represents the information content at each position in sequences. G: Glycine; A: Alanine; V: Valine; L: Leucine; I: Isoleucine; P: Proline; F: Phenylalanine; Y: Tyrosine; W: Tryptophan; S: Serine; T: Threonine; C: Cystine; M: Methionine; N: Asparagine; Q: Glutarnine; D: Asparticacid; E: Glutamicacid; K: Lysine; R: Arginine; H: Histidine.

Multiple sequence alignments of 1R-MYB, R2R3-MYB, and 3R-MYB were performed separately to characterize the DNA-binding domain of *CcMYBs* in camphor. The DNA-binding domains in the three *CcMYB* subfamilies were visualized individually using WebLogo 3 (Figure 2). The results showed that Trp (W-2) was relatively conserved, despite the large variation in the 1R-MYB amino acid residues (Figure 2A), as in previous studies [1]. In the DNA-binding domain of R2R3-MYB, there were five highly conserved Trp (W) residues (W-2, W-22, and W-42 in R2 repeats; W-81 and W-100 in R3 repeats), and the first W in R3 was often replaced by Phe (F), Ile (I), Leu (L), or Tyr (Y). In addition, some other amino acids were also relatively conserved (Figure 2B). The 3R-MYBs displayed three highly conserved and complete DNA-binding domains: R1, R2, and R3. Each R repeat contained each of the 19–20 amino acid residues, and there were 100% conserved W residues (R1: W-9, W-29, W-48; R2: W-61, W-81, W -100; R3: W-113, W-132, W-151, Figure 2C). These conserved W residues play an important role in sequence-specific DNA binding [41,42].

**Figure 3:**
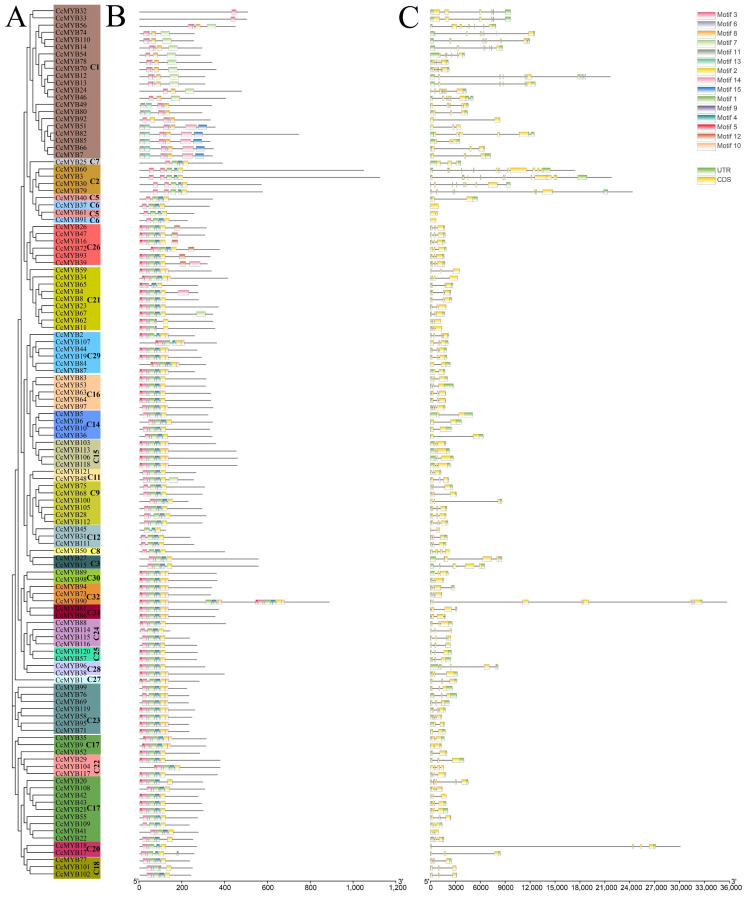
Phylogenetic relationships, motifs and exon–intron structures of *CcMYB* family members. (**A**) A phylogenetic tree of 121 *CcMYB* proteins was constructed using the neighbor-joining method. Different subgroups were indicated with different background colors and labels; (**B**) Conserved motifs of *CcMYB* proteins. Different motifs were indicated by different colored squares; (**C**) exon–intron structure of *CcMYB* genes. Exons, introns, and UTRs are indicated by yellow boxes, black lines, and green boxes, respectively.

Based on the sequence alignment results, we analyzed and visualized the conserved motifs of *CcMYBs* (Figure 3A). Figure 3B shows the distribution of the *CcMYB* motifs. A high concordance was found within each subgroup and subfamily. Subgroup C1 was identified with the 1R-MYB subfamily; the C2 subgroup was classified as 3R-MYB, and the remaining subgroups were members of R2R3-MYB (except *CcMYB65* of the C21 subgroup, which belonged to 1R-MYB). It was not difficult to find that all 121 MYB members contained motif 3. Except for 1R-MYB, which lacks motif 1, all other *CcMYBs* contain motif 1. Interestingly, although *CcMYB65* clustered with R2R3-MYB in the C21 subgroup, it lacked motif 1 as a 1R-MYB. In 3R-MYBs, all proteins had the same motif composition and shared two motif 3. In R2R3-MYBs, all the proteins contained motifs 3, 1, and 2. Across the motif distribution of all subgroups, the auxiliary motifs in the same subgroup were relatively consistent. The similarities of these motifs in the same subgroup and the differences in different subgroups suggest that *CcMYB* proteins in the same subgroup have similar functions, indicating that *CcMYBs* have undergone duplication and fragment loss during evolution, resulting in different orientations.

### 2.3. Exon–Intron Structure and Cis-Elements of CcMYB Genes

To study the similarities and differences between *CcMYBs* in *C. camphora*, we studied the *CcMYB* structure. The exon–intron structure is shown in Figure 3C. We found that the exon–intron structure of *R2R3-MYBs* was relatively consistent, mostly in the form of three exons and two introns, except that *CcMYB90* had seven introns, eight exons, and five MYB repeats. This is consistent with the previous results for other species [43]. However, the number of introns in the C1 subgroup was highly variable, ranging from 2 to 11, and the number of introns in the C3 subgroup was generally higher, including five or more introns.

**Figure 4:**
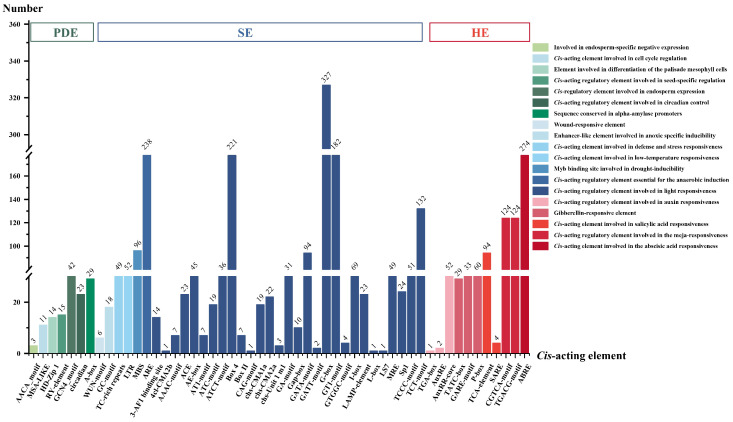
The number of various *cis*-acting elements. Different colors represent different functions. Each color group represents a different kind. The abbreviations in the above color group block, PDE: plant development-related elements; SE: stress-related elements; HE: hormone-related elements. The figure above each column represents the number of *cis*-acting elements.

Upstream of DNA, there are many promoters containing many *cis*-acting elements that are specific binding sites for regulatory proteins. We analyzed *cis*-elements in the promoter region located from −2000 to −1 bp upstream of the coding sequence of *CcMYBs*. As shown in Figure 4 and Table S4, *cis*-acting elements related to biological processes were screened for analysis, and these *cis*-acting elements were divided into 3 groups, including 19 categories according to their functions, and involving 53 *cis*-acting elements. The first group comprised plant development-related elements (PDE), the second group was of stress-responsive elements (SE), and the third group was of hormone-responsive elements (HE). The SE group had the largest number of *cis*-acting elements, with 35 *cis*-acting elements in seven categories. Light is essential for plant growth, and some studies have shown that it can boost flavonoid biosynthesis [30]. In this study, we found that light-responsive *cis*-elements were the most common type of *cis*-elements, with 1425 of the 121 *CcMYBs*, and the promoter of each *CcMYB* contained part of a light-responsive element or several light-responsive elements. Among these 53 *cis*-acting elements, the G-box, which appeared 327 times and involved 100 *CcMYBs*, was the most common *cis*-acting element.

### 2.4. Chromosome Distribution and Collinearity of CcMYB Genes

**Figure 5:**
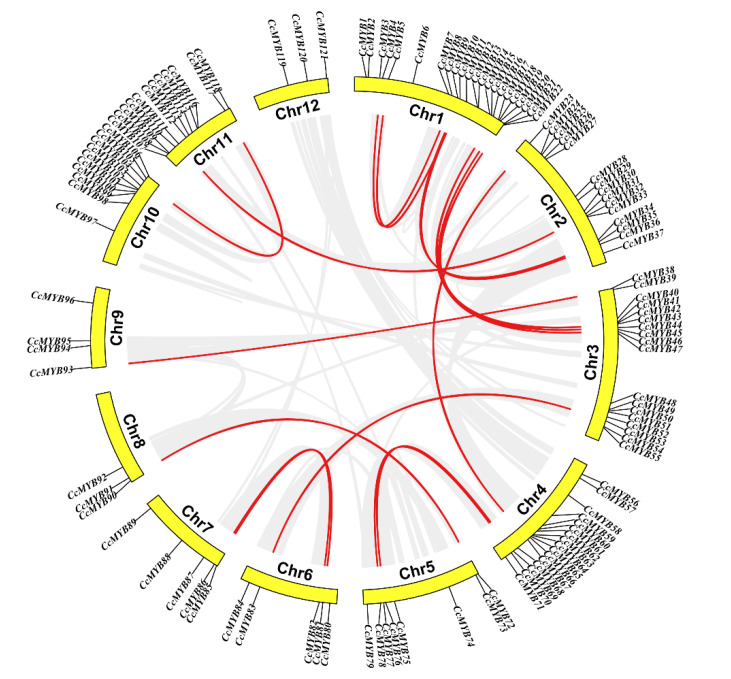
Chromosomal localization and intraspecific collinearity of *CcMYBs*. The location of the *CcMYB* gene in the camphor genome was marked on the chromosome. The middle red line represents the duplicated gene pairs for *CcMYBs*, the gray line represents the genomic duplicated gene pair, and Chr represents the chromosome.

In *C. camphora*, most of the *CcMYB* genes were located on the two ends of the 12 chromosomes, of which 121 *CcMYB* genes were randomly distributed (Figure 5). As many as 22 *CcMYBs* were distributed on chromosome 1, and the two most distant genes were more than 70 Mb apart. These 22 *CcMYBs* contained four 1R-MYBs, seven *R2R3-MYBs*, and one 3R-MYB. Among these, R2R3-MYBs involved eight subgroups, with four genes in the C17 subgroup and two genes in the C20 subgroup, which clustered with the S5 and S6 subgroups of *Arabidopsis*, respectively. However, chromosomes 8 and 12 had the fewest genes, with only three each.

We parsed the duplication events of the *CcMYBs* (Figure 5). Of these duplicated genes, only chromosome 12 had no duplicated *CcMYBs*, whereas chromosome 1 had the most duplicated *CcMYBs*, with nine different duplicated *CcMYBs*. According to these criteria, 18 genes involved in 35 *CcMYBs* (28.93%) were segmentally duplicated gene pairs. However, no tandem-duplicated genes were found, indicating that segmental duplication may be the main duplication mode of *CcMYB* family expansion and that some *CcMYBs* have undergone functional diversification and family expansion during evolution. In this study, we also calculated nonsynonymous mutations (*Ka*), synonymous mutations (*Ks*), and their ratio (*Ka*/*Ks*) to estimate selection pressure in duplicated gene pairs. The *Ks* value of 49 duplicated gene pairs were between 0.39 and 3.70, and the *Ka*/*Ks* ratios were between 0.04 and 0.52, which indicated that, during evolution, *CcMYB* duplicated gene pairs have undergone purification selection. The minimum *Ks* and maximum *Ka*/*Ks* were observed in the *CcMYB33*–*CcMYB60* pair, suggesting that these two genes may have undergone a more purifying selection.

### 2.5. Tissue-Specific Expression Profiles

**Figure 6:**
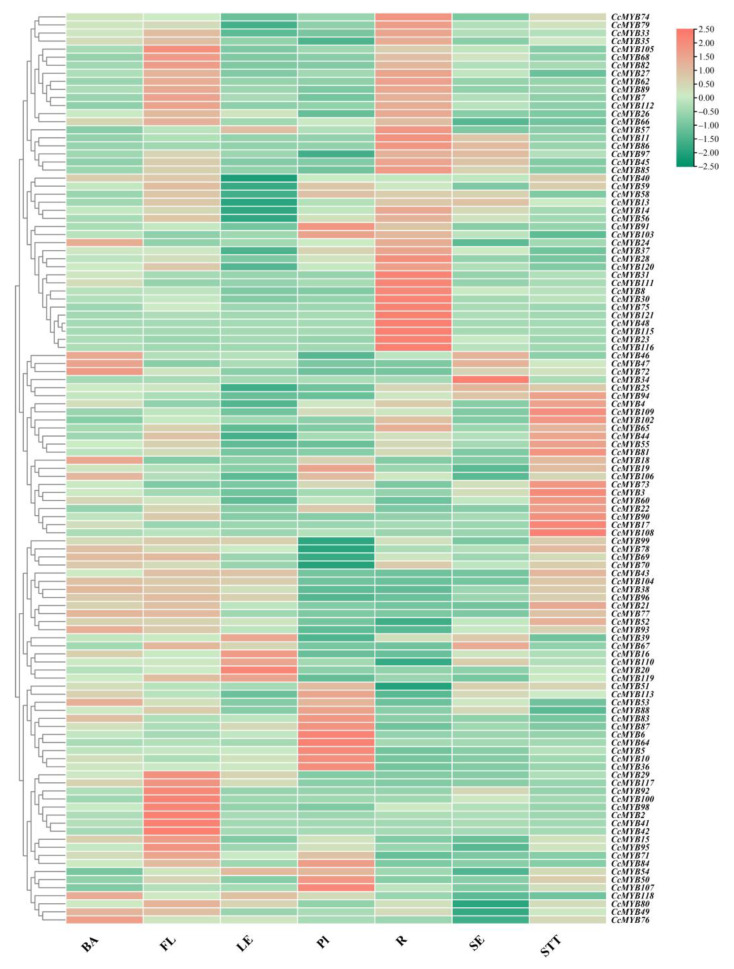
The expression of *CcMYBs* in different tissues by RNA-Seq. Expression of different *CcMYBs* in phloem (BA), flower (FL), leaf (LE), xylem (Pl), root (R), seed (SE), and stem (STT), with the vertical axis showing gene names and the horizontal axis showing different tissues. The different colors correspond to fold changes in log2, with green and red indicating downregulation and upregulation, respectively.

To investigate the spatial expression profiles of *CcMYBs* in *C. camphora*, we analyzed the expression of *CcMYBs* in the phloem (BA), flower (FL), leaf (LE), xylem (Pl), root (R), fruit (SE), and stem (STT) in ‘Gantong 1’. In the transcription data of seven tissues of *C. camphora*, four *CcMYBs* (*CcMYB1*, *CcMYB9*, *CcMYB61*, and *CcMYB114*) were not expressed in any tissue of *C. camphora*, which may be because they were pseudogenes or not expressed in our selected samples. The transcription levels of *CcMYB37*, *CcMYB66*, and *CcMYB80* were higher in all tissues than most of the other genes (Table S3). Cluster analysis of the expressed data was performed and a heat map of *CcMYB* expression was generated, as shown in Figure 6. Thirty *CcMYBs* had higher FPKM values in the roots than in the other tested tissues, but only four *CcMYBs* had the highest expression levels in fruits. To explore whether *CcMYBs* in the C17, C20, and C22 subgroups were also tissue-specific, the expression of 16 *CcMYBs* were analyzed that did not count *CcMYB9* because of the lack of expression in each tissue. Eight *CcMYBs* were found to have higher expression levels in the stem than in other tissues, and there were two *CcMYBs* with the highest expression in the phloem, suggesting that *C. camphora* flavonoids may mainly accumulate in the bark.

**Figure 7:**
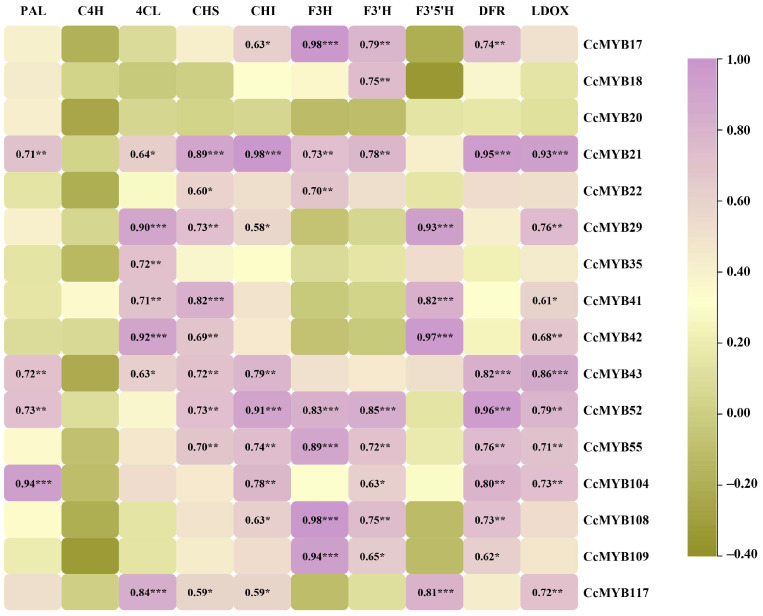
Correlation between *CcMYB* genes expression and the expression of structural genes in the flavonoid biosynthesis pathway. PAL, Phenylalanine ammonia lyase; C4H, Cinnamate 4-hydroxylase; 4CL, 4-coumarate:coenzyme A ligase; CHS, Chalcone synthase; CHI, Chalcone isomerase; F3H, Flavanone 3-hydroxylase; F3′H, Flavonoid 3′-hydroxylase; F3′5′H, Flavonoid 3′,5′-hydroxylase; DFR, Dihydroflavonol 4-reductase; LDOX, Leucoanthocyanidin dioxygenase. ***: *p* < 0.001, **: *p* < 0.01, *: *p* < 0.05.

**Figure 8:**
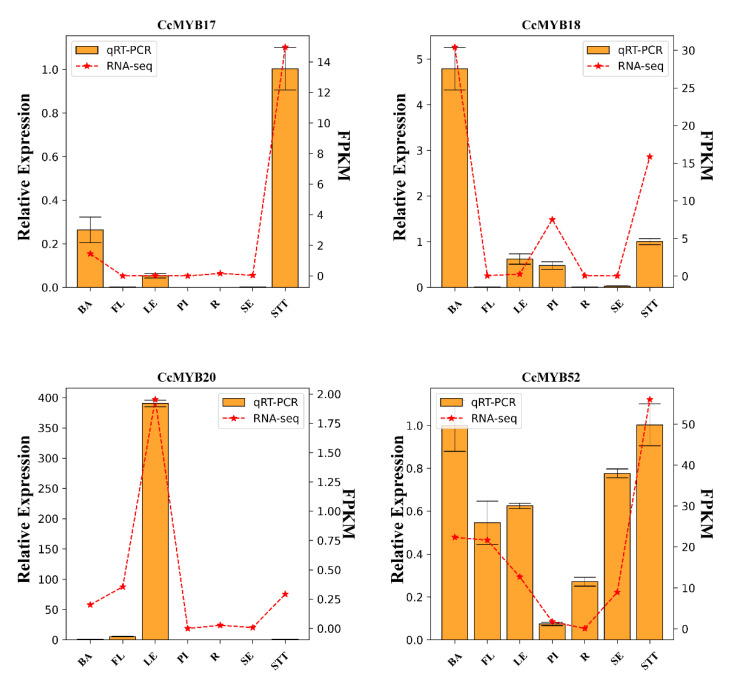
Expression validation of *MYB* genes related to flavonoid synthesis in different tissues and qRT-PCR and RNA-Seq results for four *CcMYB* genes in seven tissues of *C. camphora*. The abscissa represents seven tissues, in the following order: phloem (BA), flower (FL), leaf (LE), xylem (Pl), root (R), seed (SE), and stem (STT). The left ordinate represents the relative expression and the right ordinate represents FPKM. Orange bars: Expression data were normalized to *CcActin* gene expression levels, and the amount of expression in stems was normalized to “1”. Mean expression values were calculated from three replicates, and the vertical bars indicate the standard error of the mean. Red line graph: RNA-Seq expression results.

Expression correlation analysis of 16 *CcMYBs* in the C17, C20, and C22 subgroups showed that these *CcMYBs* were significantly associated with structural genes involved in flavonoid synthesis (Figure 7). *CcMYB21* and *CcMYB52* were significantly associated with the majority of structural genes in this pathway. Some genes, such as *CcMYB21*, *CcMYB43*, and *CcMYB52*, were significantly correlated with the DFR and LDOX, which are the late biosynthetic genes (LBGs) of the anthocyanin synthesis pathway. Four *CcMYBs* were selected to study their expression profiles in seven different tissues by quantitative real-time PCR (Figure 8). The results showed that their expression profiles were consistent with transcriptome data and showed notable tissue specificity. Quantitative data showed that these *CcMYBs* were markedly downregulated in the flowers, xylem, and roots. The expression levels of *CcMYB20* were higher in the leaves than in the other six tested tissues and were hardly expressed in the seeds and xylem, while other *CcMYBs* had a relatively high expression in the stems of *C. camphora*.

## 3. Discussion

The MYB transcription factor family is a protein superfamily with a large number of members and diverse functions. Based on the whole genome, ninety-six R2R3-MYBs, twenty-one 1R-MYBs, and four 3R-MYBs were identified and no 4R-MYBs were identified. The different amounts of *CcMYBs* of the same species identified in other studies can be attributed to advancements in technology and different identification standards; however, the fact that R2R3-MYB dominates the plant MYB family has not changed. In contrast, the proportions of 3R-MYB and 4R-MYB were previously found to be very small; the same results were obtained in this study. Further research on the DNA-binding domain of R2R3-MYB found that there were five highly conserved Trp (W) residues but the first W in R3 was often replaced by Phe (F), Ile (I), Leu (L), or Tyr (Y) and some other amino acids were also relatively conserved, suggesting that the repeats of the DNA-binding domains of 121 *CcMYBs* shared characteristics with those of the other species [43,44], while there were nine 100% conserved W residues in 3R-MYB. The auxiliary motifs of MYB TFs are also important features for MYB classification [41,45]. A previous report study has put forward insight on the composition and evolutionary history of plant *R2R3-MYBs* based on the analysis of multiple levels and aspects, such as auxiliary motifs and phylogenetic analysis [46]. In this study, across the motif distribution of all subgroups, the characteristics of auxiliary motifs suggest that *CcMYB* proteins in the same subgroup have similar functions, indicating that *CcMYBs* have undergone duplication and fragment loss during evolution, resulting in different orientations. Chromosome mapping showed that 121 *CcMYBs* were unevenly distributed on 12 chromosomes. Tandem and segmental duplication events are thought to be the main reasons for the expansion of gene families in the genome [47]. A previous report found that *C. camphora* has undergone two genome-wide duplication events, and 1110 gene families have expanded [33]. As suggested in another previous report on analyzing the duplication events of genes in camphor trees, both of the two duplication events promoted the expansion of the *TPS* gene family in the *C. camphora* genome significantly [33,48]. Therefore, we explored duplication events in the *CcMYB* gene family. Segmental duplication events occurred in 18 gene pairs on 11 chromosomes. Similarly, many segmental duplication events occurred in potato (*Solanum tuberosum* L.), Pistacia chinensis, poplar, and other species [46,49,50,51], implying that segmental duplication events may be vital factors in the expansion of the *MYB* TF family.

The R2R3-MYB of *Arabidopsis* was divided into 23 subgroups according to the auxiliary motif [41]. It is well known that there are large differences between herbaceous and woody plants during the evolutionary process, but only a few herbaceous species have been compared in previous studies. In addition, there were some *MYB* genes that had not been classified, so this classification method is not universal, and there is no unified classification method for R2R3-MYB [46,52,53]. Referring to the grouping of *Arabidopsis* and poplar, MYBs were roughly divided into 32 subgroups according to the protein sequence alignment results, of which four subgroups did not contain *CcMYBs*. In this study, we also classified some genes that were not grouped in previous reports according to their later reported functions. It has been reported that AtMYB5, which may be homologous to TT2 (AtMYB123) in terms of function, can also form MBW complexes with bHLH and WD40 [54], so we combined TT2 and AtMYB5 into one group. Although we did not further the understanding of group 1R-MYB, there have been reports on the classification of 1R-MYB [31]. As shown in Figure 2, gene structures and conserved motifs of *CcMYBs* in the same subgroup were mostly similar, which strongly confirmed the reliability of our classification of *CcMYBs*.

**Figure 9:**
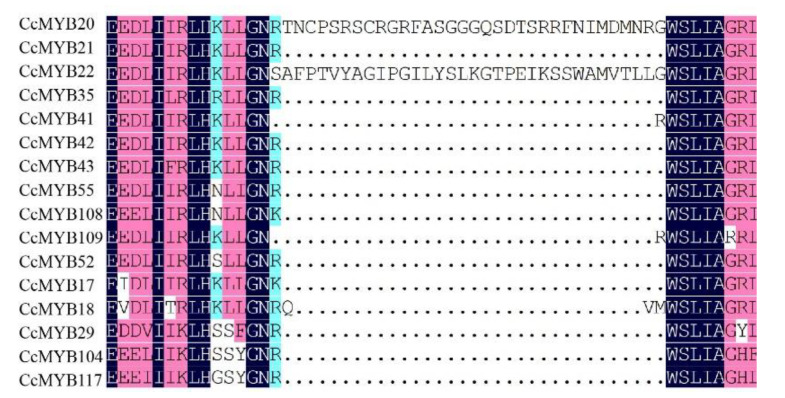
Multiple sequence alignment of R3 structures in subgroups C17, C20, and C22.

There is ample evidence proving that MYB TFs play a crucial role in the direct or indirect response to stress. AtMYB72 affects signaling pathways induced by these beneficial microorganisms [55]. AtMYB15 is involved in the cold regulation of C-repeat binding transcription factor genes and in the development of freezing tolerance [56]. To date, some studies have focused on the functions of MYBs in flavonoid synthesis because flavonoids are widely involved in abiotic stress and floral pigmentation in higher plants. Interestingly, in this work, it was found that the C22, C17, and C20 subgroups of the camphor tree clustered together with S7, S5, and S6 subgroups in *A. thaliana*, respectively. It has been confirmed that S7 increases flavonoid biosynthesis in *Arabidopsis* [40], S5 regulates pro-anthocyanidin synthesis in the seed coat [41], and S6 regulates anthocyanin biosynthesis [42]. In addition, the results of the correlation analysis between 16 *CcMYBs*, including *CcMYB17* and *CcMYB18*, and structural genes in the flavonoid biosynthesis pathway revealed that they were significantly correlated. F3H, F3’5’H, and F3’H lead to the synthesis of different anthocyanins, which are catalyzed by DFR and ANS (anthocyanidin synthase)/LDOX, resulting in the formation of pelargonidin, cyanidin, and delphinidin, respectively [57,58]. Moreover, correlation analysis showed that some of these 16 genes were significantly correlated with DFR and LDOX (Figure 8). Sequence alignment analysis revealed that the R3 structures of the 16 *CcMYBs*, except for the C22 subgroup (which clustered a branch with the *Arabidopsis* S7 subgroup), contained the motif LX_2_X_3_LX_6_LX_3_R (*CcMYB20* and *CcMYB22* were incompletely consistent) [59], which can bind to bHLH (Figure 9). This observation is consistent with findings in *Arabidopsis*, suggesting that these genes have the potential to form MBW complexes [54] (Figure 3). Four genes (*CcMYB17*, *CcMYB18*, *CcMYB20*, and *CcMYB52*) were selected for real-time quantification in different tissues, and the results showed significant differential tissue expression. Some MYBs in *Hypericum perforatum, Dendrobium catenatum*, and tea plants (*Camellia sinensis*) showed similar results [60,61,62]. Although the expression patterns of these genes are different, they all seem to have relatively high expression in the phloem and stem, which is mutually confirmed by the previous results that the stem of ‘Gantong 1′ is red and the bark of cinnamon has a unique scent [63,64]. In addition, MYB TFs are useful for plant development, cell shape, and tissue morphogenesis [26,65].

This study revealed the basic characteristics of the *CcMYB* gene family and predicted the related *CcMYBs* that may regulate the flavonoid synthesis pathway, which provides valuable information for studying the synthesis of secondary metabolites and the growth and development of *C. camphora*.

## 4. Materials and Methods

### 4.1. Plant Materials

A 3-year-old clonal cutting seedling of ‘Gantong 1′, which is a new national plant variety of camphor tree cultivated by the Jiangxi Academy of Sciences, was used as the experimental material. Compared with common camphor, the content of anthocyanins, such as pelargonidin, cyanidin, and peonidin, in ‘Gantong 1’ demonstrated a prominent increase [63]. All the materials were planted in Huangma Township, Nanchang City, Jiangxi Province, China. Roots, flowers, phloem, leaves, xylem, and stem were collected in April 2020, whereas fruits were collected in November 2020 for a total of seven tissues. Three biological replicates were used for each experiment.

### 4.2. Bioinformatics Analysis of MYB Transcription Factor Genes in C. comphora

The MYB domain seed file (PF00249) was downloaded from the Pfam 31.0 database, then HMMER 3.2.1 software was used to create a profile hidden Markov model (HMM), before the candidate MYB protein sequence of *C. camphora* was obtained [66,67]. Based on the genome data of *C. camphora* published previously, the MYB domains of candidate *C. camphora* were checked by setting an E value ≤ 1 × 10^−10^, alignment length >100, and alignment rate >50%. After excluding redundant sequences and incomplete sequences, NCBI-Conserved Domain Data (CDD) and SMRT online websites were used to screen out MYB members in *C. camphora*.

Amino acid residues, molecular weight (MW), isoelectric point (PI), stability coefficient, and grand average of hydropathicity (GRAVY) of MYB protein in *C. camphora* were identified using the ProtParam tool on the ExPASy online website [68].

The MYB protein sequences of *A. thaliana* and poplar were queried from the TAIR and JGI databases, respectively. MYB proteins in *C. camphora* and *P. trichocarpa* were named according to their chromosomal locations, whereas those in *Arabidopsis* were named according to the TAIR database. The conserved domains of MYB proteins from *C. camphora*, *Arabidopsis*, and *P. trichocarpa* were aligned multiple times with ClustalW. Phylogenetic trees were constructed using the neighbor-joining method of MEGA X. Using the bootstrap test method, the number of replicates was set to 1000 [69]. The phylogenetic tree was annotated and visualized using the ITOL v6.6 online tool.

Python was used to analyze the exon–intron of the gene structure, and TBtools v1.0987663 was used to visualize it [70]. The *cis*-acting elements of the *CcMYB* promoter region were analyzed using the PlantCare website.

The conserved motifs of *CcMYB* were analyzed according to their amino acid sequences using the MEME program. The motif site distribution in the sequences was 0 or 1 per sequence. The number of motifs was set to 15, and the width ranged from six to 50 amino acids. Only the motif with an E < 0.05 was retained for further analysis. The results were visualized and enhanced using TBTools. The amino acid sequences of R1, R2, and R3 repeats in *CcMYB* were extracted, and ClustalW was used for multiple sequence alignment. Sequence logos were generated using WebLogo3 [71].

TBtools were used to construct a Circos map that displays the relationship between chromosomes and the position and relative distance of genes on chromosomes. Confirmation of *CcMYB* gene duplication was based on two criteria: (a) the length of the shorter sequence exceeding 70% of the longer sequence, and (b) the similarity between the two aligned sequences being greater than 70% [71,72]. All *CcMYBs* with duplicated segments were selected to generate a resonance map with putative duplicated gene pairs connected by connecting lines.

The paralogous gene pair of *C. camphora* was defined as a sequence length >300 bp and homology ≥50%. The amino acid sequences of these gene pairs were aligned using Clustal W. Ks and Ka substitution rates were calculated using the aligned file and corresponding coding nucleotide sequences using the PAL2NAL program [73].

### 4.3. Gene Expression Analysis

We analyzed the expression profiles of *CcMYBs* in different tissues of *C. camphora* using transcriptome data. Transcriptome data of all seven *C. camphora* tissues were obtained from our laboratory and uploaded to the NCBI database [33]. FPKM for each gene was calculated based on the length of the gene and mapped to the read count for that gene. The expression data of *CcMYBs* in seven *C. camphora* tissues were extracted and analyzed using Python.

Total RNA was extracted according to the instructions of the RNAprep Pure Plant Plus Kit (polysaccharides and polyphenolics-rich) (Tiangen Biotech, Beijing, China). RNA degradation and contamination were monitored on a 1% agarose gel. Total RNA was reverse-transcribed using a 5× PrimeScript RT Master Mix (TaKaRa). Quantitative primers for the selected *CcMYB* gene were designed using Beacon Designer 8 software (Table S1). Semi-quantitative PCR experiments were performed to verify the primer specificity. The fluorescent dye used in the real-time quantitative experiment was PowerUp^TM^ SYBR^TM^ Green Master Mix (TaKaRa), and analysis was performed using the Applied Biosystems ViiA 7 system. *CcActin* was used as the reference gene for qRT-PCR. The relative expression levels of the *CcMYBs* were calculated using the 2^−ΔΔct^ method and TBtools was used to construct a heatmap to visualize the results. Statistical differences were determined by one-way ANOVA variance using Python.

## Figures and Tables

**Table 1:** Nomenclature and classification of MYB family genes in *C. camphora*.

Gene ID	MYB Name	No. of Domains	AT Hit	Gene ID	MYB Name	No. of Domains	AT Hit
Ccam01T000258.1	*CcMYB1*	2	AtMYB29	Ccam04T001684.1	*CcMYB62*	2	AtMYB84
Ccam01T000296.1	*CcMYB2*	2	AtMYB40	Ccam04T001846.1	*CcMYB63*	2	AtMYB26
Ccam01T000688.1	*CcMYB3*	3	AtMYB3R1	Ccam04T001846.2	*CcMYB64*	2	AtMYB26
Ccam01T000754.1	*CcMYB4*	2	AtMYB38	Ccam04T001876.1	*CcMYB65*	1	AT2G36890
Ccam01T000934.1	*CcMYB5*	2	AtMYB46	Ccam04T001898.1	*CcMYB66*	1	AT5G47390
Ccam01T001469.1	*CcMYB6*	2	AtMYB46	Ccam04T001984.1	*CcMYB67*	2	AtMYB36
Ccam01T001922.1	*CcMYB7*	1	AT5G47390	Ccam04T002420.1	*CcMYB68*	2	AtMYB116
Ccam01T001979.1	*CcMYB8*	2	AtMYB38	Ccam04T002616.1	*CcMYB69*	2	AtMYB4
Ccam01T002141.1	*CcMYB9*	2	AtMYB5	Ccam04T002681.1	*CcMYB70*	part	AT3G24120
Ccam01T002197.1	*CcMYB10*	2	AtMYB46	Ccam04T002944.1	*CcMYB71*	2	AtMYB4
Ccam01T002391.1	*CcMYB11*	2	AtMYB84	Ccam05T000046.1	*CcMYB72*	2	AtMYB94
Ccam01T002796.1	*CcMYB12*	part	AT3G24120	Ccam05T000101.1	*CcMYB73*	2	AtMYB93
Ccam01T002796.2	*CcMYB13*	part	AT3G24120	Ccam05T000964.2	*CcMYB74*	part	AT2G01060
Ccam01T003262.1	*CcMYB14*	part	AT2G01060	Ccam05T001874.1	*CcMYB75*	2	AtMYB116
Ccam01T003298.1	*CcMYB15*	2	AtMYB33	Ccam05T002107.1	*CcMYB76*	2	AtMYB4
Ccam01T003334.1	*CcMYB16*	2	AtMYB60	Ccam05T002146.1	*CcMYB77*	2	AtMYB66
Ccam01T003511.1	*CcMYB17*	2	AtMYB113	Ccam05T002218.1	*CcMYB78*	part	AT3G24120
Ccam01T003512.1	*CcMYB18*	2	AtMYB113	Ccam05T002668.1	*CcMYB79*	3	AtMYB3R5
Ccam01T003530.1	*CcMYB19*	2	AtMYB43	Ccam06T000233.1	*CcMYB80*	1	AT5G61620
Ccam01T003681.1	*CcMYB20*	2	AtMYB123	Ccam06T000471.1	*CcMYB81*	2	AtMYB9
Ccam01T003682.1	*CcMYB21*	2	AtMYB123	Ccam06T000579.1	*CcMYB82*	1	AT5G47390
Ccam01T003684.1	*CcMYB22*	2	AtMYB123	Ccam06T001498.1	*CcMYB83*	2	AtMYB103
Ccam02T000146.1	*CcMYB23*	2	AtMYB36	Ccam06T001794.1	*CcMYB84*	2	AtMYB42
Ccam02T000623.1	*CcMYB24*	0	AT3G04030	Ccam07T000059.1	*CcMYB85*	1	AT5G47390
Ccam02T000634.1	*CcMYB25*	2	AtMYB105	Ccam07T000142.1	*CcMYB86*	2	AtMYB9
Ccam02T000715.1	*CcMYB26*	2	AtMYB60	Ccam07T000603.1	*CcMYB87*	2	AtMYB42
Ccam02T000749.1	*CcMYB27*	2	AtMYB65	Ccam07T001311.1	*CcMYB88*	2	AtMYB58
Ccam02T001376.1	*CcMYB28*	2	AtMYB78	Ccam07T002058.1	*CcMYB89*	2	AtMYB102
Ccam02T001462.2	*CcMYB29*	2	AtMYB111	Ccam08T000090.1	*CcMYB90*	5	AtMYB93
Ccam02T001819.1	*CcMYB30*	3	AtMYB3R5	Ccam08T000196.1	*CcMYB91*	2	AtMYB73
Ccam02T001906.1	*CcMYB31*	2	AtMYB59	Ccam08T000630.1	*CcMYB92*	2	AT3G10580
Ccam02T001944.1	*CcMYB32*	1	AT1G15720	Ccam09T000028.1	*CcMYB93*	2	AtMYB30
Ccam02T001944.2	*CcMYB33*	1	AT1G15720	Ccam09T000765.1	*CcMYB94*	2	AtMYB93
Ccam02T002770.1	*CcMYB34*	2	AtMYB35	Ccam09T000873.1	*CcMYB95*	2	AtMYB4
Ccam02T002809.1	*CcMYB35*	2	AtMYB5	Ccam09T001430.1	*CcMYB96*	2	AtMYB17
Ccam02T002880.1	*CcMYB36*	2	AtMYB46	Ccam10T000428.1	*CcMYB97*	2	AtMYB67
Ccam02T003202.1	*CcMYB37*	2	AtMYB44	Ccam10T000876.1	*CcMYB98*	2	AtMYB102
Ccam03T000003.1	*CcMYB38*	2	AtMYB106	Ccam10T001218.1	*CcMYB99*	2	AtMYB4
Ccam03T000033.1	*CcMYB39*	2	AtMYB94	Ccam10T001249.1	*CcMYB100*	2	AtMYB21
Ccam03T001088.1	*CcMYB40*	2	AtMYB109	Ccam10T001299.1	*CcMYB101*	2	AtMYB23
Ccam03T001159.1	*CcMYB41*	2	AtMYB5	Ccam10T001299.2	*CcMYB102*	2	AtMYB23
Ccam03T001160.1	*CcMYB42*	2	AtMYB123	Ccam10T001421.1	*CcMYB103*	2	AtMYB55
Ccam03T001161.1	*CcMYB43*	2	AtMYB123	Ccam10T001628.1	*CcMYB104*	2	AtMYB111
Ccam03T001249.1	*CcMYB44*	2	AtMYB20	Ccam10T001729.1	*CcMYB105*	2	AtMYB78
Ccam03T001287.1	*CcMYB45*	2	AtMYB59	Ccam10T001828.1	*CcMYB106*	2	AtMYB61
Ccam03T001320.1	*CcMYB46*	part	AT3G04030	Ccam11T000037.1	*CcMYB107*	2	AtMYB43
Ccam03T001354.1	*CcMYB47*	2	AtMYB60	Ccam11T000138.1	*CcMYB108*	2	AtMYB123
Ccam03T002316.1	*CcMYB48*	2	AtMYB71	Ccam11T000141.1	*CcMYB109*	2	AtMYB5
Ccam03T002477.1	*CcMYB49*	1	AT5G47390	Ccam11T000460.1	*CcMYB110*	part	AT2G01060
Ccam03T002495.1	*CcMYB50*	2	AtMYB33	Ccam11T000608.1	*CcMYB111*	2	AtMYB59
Ccam03T002497.1	*CcMYB51*	1	AT5G47390	Ccam11T000931.1	*CcMYB112*	2	AtMYB78
Ccam03T002631.1	*CcMYB52*	2	AtMYB5	Ccam11T001000.1	*CcMYB113*	2	AtMYB61
Ccam03T002754.1	*CcMYB53*	2	AtMYB103	Ccam11T001081.1	*CcMYB114*	2	AtMYB15
Ccam03T002940.1	*CcMYB54*	part	AT3G24120	Ccam11T001150.1	*CcMYB115*	2	AtMYB15
Ccam03T002949.1	*CcMYB55*	2	AtMYB123	Ccam11T001151.1	*CcMYB116*	2	AtMYB15
Ccam04T000335.1	*CcMYB56*	part	AT5G29000	Ccam11T001744.1	*CcMYB117*	2	AtMYB12
Ccam04T000453.1	*CcMYB57*	2	AtMYB15	Ccam11T001870.1	*CcMYB118*	2	AtMYB61
Ccam04T000811.1	*CcMYB58*	2	AtMYB4	Ccam12T000355.1	*CcMYB119*	2	AtMYB4
Ccam04T001106.1	*CcMYB59*	2	AtMYB80	Ccam12T000791.1	*CcMYB120*	2	AtMYB15
Ccam04T001450.1	*CcMYB60*	3	AtMYB3R4	Ccam12T001449.1	*CcMYB121*	2	AtMYB71
Ccam04T001597.1	*CcMYB61*	2	AtMYB109				

AT: *Arabidopsis thaliana*.

## Data Availability

All the data are shown in the main manuscript and in the Supplementary Materials.

## Supplementary Materials

The following supporting information can be downloaded at: https://www.mdpi.com/article/10.3390/ijms232214279/s1.
